# The Weakened Relationship Between Prestimulus Alpha Oscillations and Response Time in Older Adults With Mild Cognitive Impairment

**DOI:** 10.3389/fnhum.2020.00048

**Published:** 2020-03-12

**Authors:** Yiqi Chen, Hao He, Pengfei Xu, Jing Wang, Yuehong Qiu, Wei Feng, Yuejia Luo, Li Hu, Qing Guan

**Affiliations:** ^1^Center for Brain Disorders and Cognitive Sciences, Shenzhen University, Shenzhen, China; ^2^School of Psychology, Shenzhen University, Shenzhen, China; ^3^Shenzhen-Hong Kong Institute of Brain Science-Shenzhen Fundamental Research Institutions, Shenzhen, China; ^4^Center for Neuroimaging, Shenzhen Institute of Neuroscience, Shenzhen, China; ^5^School of Marxism, Jilin Medical University, Jilin, China; ^6^CAS Key Laboratory of Mental Health, Institute of Psychology, Chinese Academy of Sciences, Beijing, China

**Keywords:** mild cognitive impairment, preparatory attention, prestimulus alpha oscillations, response time, machine learning

## Abstract

**Background:** Prestimulus alpha oscillations associated with preparatory attention have an impact on response time (RT). However, little is known about whether there is a deficit in the relationship between prestimulus alpha oscillations and RT in older adults with mild cognitive impairment (MCI).

**Method:** We collected electroencephalography (EEG) data from 28 older adults with MCI and 28 demographically matched healthy controls (HCs) when they were performing an Eriksen flanker task. For each participant, single-trial prestimulus alpha power was calculated for combinations of congruency (congruent vs. incongruent) and response speed (fast vs. slow).

**Result:** Statistical analysis indicated that prestimulus alpha power was significantly lower for fast trials than slow trials in HCs but not in older adults with MCI. The Fisher’s *z* scores of the within-subject correlation coefficients between single-trial prestimulus alpha power and RT were significantly larger in HCs than in older adults with MCI. In addition, machine learning analyses indicated that prestimulus alpha power and its correlation with RT could serve as features to distinguish older adults with MCI from HCs and to predict performance on some neuropsychological tests.

**Conclusion:** The reduced correlation between prestimulus alpha activity and RT suggests that older adults with MCI experience impaired preparatory attention.

## Introduction

Mild cognitive impairment (MCI) refers to an intermediate stage between normal cognitive decline due to aging and a more severe decline due to dementia (Albert et al., 2011; Petersen et al., 2018). One of the main characteristics of MCI is impaired preparatory attention (Silveri et al., 2007), which entails impairment in one’s ability to continuously mentally prepare for incoming events to be able to cope with them (Perry, 1999). Previous studies have demonstrated that participants with MCI show impaired preparatory attention in choice reaction time tasks and cued target detection tasks (Levinoff et al., 2005; Tales et al., 2005, 2011). In these tasks with cues, participants with MCI, compared to those with normal cognition, were less sensitive to prestimulus cues that induced a decrease in the power of prestimulus alpha oscillations and a shorter response time (RT; van den Berg et al., 2014). However, in paradigms without cues, the influence of preparatory attention on RT in participants with MCI remains unknown.

Preparatory attention is associated with a constantly changing mental state that is reflected by fluctuating alpha oscillations embedded in spontaneous electroencephalography (EEG) signals (Haegens et al., 2011; Hanslmayr et al., 2013; Battistoni et al., 2017). Prestimulus alpha oscillations influence poststimulus processes and stimulus-evoked brain responses (Başar, 2012; Slagter et al., 2016; Tu et al., 2016). Previous studies have indicated that a lower power in prestimulus alpha oscillations is associated with higher subjective ratings of preparatory attention (Worden et al., 2000; Klimesch, 2012) and perceptual awareness (Baumgarten et al., 2016; Benwell et al., 2017), as well as with better perceptual acuity (Baumgarten et al., 2016; Roberts et al., 2014) and executive control (Mazaheri et al., 2011; Shou et al., 2015). In addition, a lower prestimulus alpha power is associated with faster responses in both monkeys and humans (Zhang et al., 2008; Bompas et al., 2015; van den Berg et al., 2016). However, little research has examined alterations in the relationship between prestimulus alpha activity and RT in older adults with MCI.

In the present study, we aimed to investigate whether there was a deficit in the relationship between preparatory attention and RT in older adults with MCI, in terms of the electrophysiological correlate of preparatory attention–prestimulus alpha-band oscillations. The weakened association between preparatory attention and RT in older adults with MCI, compared to those with healthy cognitive functions, is likely to be represented by: (1) a smaller difference in prestimulus alpha power between trials with fast and slow response speed; and (2) a reduced positive correlations between prestimulus alpha power and RT.

During an Eriksen flanker task (Eriksen and Eriksen, 1974), EEG signals were recorded from older adults with MCI and demographically matched healthy controls (HCs). For each participant, all trials in the congruent and incongruent conditions were divided into fast and slow bins based on single-trial RTs. The difference in the prestimulus alpha power between the two bins was compared in older adults with MCI and HCs. In addition, within-subject correlations between single-trial prestimulus alpha power and RT were compared between older adults with MCI and HCs. Finally, we applied support vector machine (SVM) analysis to investigate whether prestimulus alpha power and its correlation with RT could distinguish older adults with MCI from HCs and predict performance on neuropsychological tests.

## Methods

### Participants

Older adults with MCI and those with normal cognition were recruited from communities in Shenzhen City, China. The demographic information of the participants was collected using a questionnaire designed for this study. The Chinese version of the Mini-Mental State Examination (MMSE; Folstein et al., 1975), a combined version of the Physical Self-Maintenance Scale, and the Instrumental Activities of Daily Living Scale (Lawton and Brody, 1969) were administered. Participants with an MMSE score lower than 24, impaired activities of daily living (ADLs), or impaired instrumental activities of daily living (IADLs) were excluded from the study to avoid the potential confounding effect due to dementia or impaired ADLs/IADLs.

To identify participants with MCI, 10 neuropsychological tests were performed to assess the following five cognitive domains: (1) memory, assessed using the Auditory Verbal Learning Test (AVLT, using the AVLT delayed recall score and the AVLT total score; Schmidt, 1996) and the Rey–Osterrieth Complex Figure Recall Test (ROCFT Recall; Shin et al., 2006); (2) executive function, assessed using the Trail Making Test Part B (TMT-B; Gordon, 1972) and the Stroop test (ST; Koss et al., 1984); (3) attention, assessed using the Trail Making Test Part A (TMT-A; Gordon, 1972) and the Symbol Digit Modalities Test (SDMT; Smith, 1982); (4) language, assessed using the Category Verbal Fluency Test (CVFT; Mok et al., 2004) and the Boston Naming Test (BNT; Knesevich et al., 1986); and (5) visuospatial ability, assessed using the Rey–Osterrieth Complex Figure Copy Test (ROCFT Copy; Shin et al., 2006) and the Clock Drawing Test (CDT; Shulman, 2000). Cognitive dysfunction in a domain was defined as a participant’s score in the domain being lower than the 1.5-SD cut off [i.e., 1.5 *SD* lower than the grand mean of the norms (Li et al., 2013)]. According to Petersen’s criteria of MCI (Petersen, 2004), a participant was identified as having MCI if he/she showed cognitive dysfunction in any of the five cognitive domains assessed.

A total of 60 older adults participated in the experiment, of which 30 were older adults with MCI and 30 were HCs. Four participants were excluded because of low accuracy in the task or poor quality of EEG signals. As a result, 28 older adults with MCI (13 females and 15 males; mean age = 67.8 years; age range, 62.0–80.5 years; years of education = 8.2 years) and 28 demographically matched HCs (13 females and 15 males; mean age = 67.5 years; age range, 61.8–76.1 years; years of education = 9.3 years) were included in further analyses. Details of the participants’ demographic information and neuropsychological performance are presented in Table 1. All participants were right-handed and had normal or corrected-to-normal vision. The protocol was approved by the Institutional Review Board (IRB) of Shenzhen University. Written informed consents were obtained from all participants.

**Table 1:** The demographic information and the performance on neuropsychological tests in older adults with mild cognitive impairment (MCI) and healthy controls (HCs).

	MCI	HC	*t*	*p*
Age	67.84 (4.03)	67.51 (4.02)	0.31	0.76
Sex	0.54 (0.51)	0.54 (0.51)	0	1.00
Education	8.21 (2.87)	9.33 (2.49)	−1.57	0.12
MMSE	26.64 (1.50)	28.39 (1.20)	−4.83	<0.001
Auditory Verbal Learning Test (Delayed recall)	3.36 (2.72)	6.14 (1.96)	−4.39	<0.001
Auditory Verbal Learning Test (Total score)	19.36 (9.80)	29.50 (6.70)	−4.52	<0.001
Rey–Osterrieth Complex Figure Test (Delayed recall)	8.46 (6.70)	15.75 (5.83)	−4.35	<0.001
Trail Making Test (Part B)	230.46 (72.10)	153.25 (58.21)	4.41	<0.001
Stroop Test	95.89 (33.46)	83.64 (24.03)	1.57	0.12
Trail Making Test (Part A)	71.75 (22.04)	53.36 (14.55)	3.69	<0.001
Symbol Digit Modalities Test	25.75 (11.80)	34.92 (10.31)	−3.10	<0.01
Category Verbal Fluency Tests	15.00 (4.82)	18.79 (5.49)	−2.74	<0.01
Boston Naming Test	20.11 (3.70)	24.75 (3.11)	−5.09	<0.001
Rey–Osterrieth Complex Figure Test (Copy)	29.82 (6.35)	33.71 (3.43)	−2.85	<0.01
Clock Drawing Test	21.46 (6.27)	25.75 (4.46)	−2.90	<0.01

*Data are shown as mean (standard deviation)*.

## Materials

**Figure 1:**
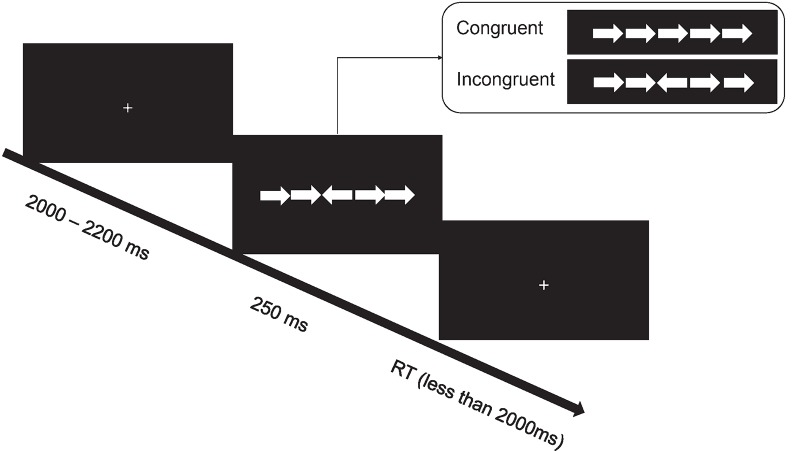
The schematic diagram of the Eriksen flanker task.

In the Eriksen flanker task, stimuli consisted of a row of five horizontal white arrows (one central target and four flankers), pointing to either the left or the right, against a black background (Figure 1). We manipulated the congruency of arrow direction: congruent (the central target pointed to the same direction as the flankers) vs. incongruent (the central target pointed to the opposite direction as that of the flankers). At the beginning of each trial, there was a fixation cross presented for 2–2.2 s. Following this fixation period, stimuli (i.e., a row of five horizontal arrows) were presented for 250 ms. Afterward, a fixation cross with the same size to previous one was presented for 2 s, and the current trial ended when a response was made. Participants were required to indicate the direction of the target arrow by pressing a mouse button. The left button indicated the left direction, and the right button indicated the right direction. A practice session with 30 trials included was performed before the test session with 60 congruent and 60 incongruent trials that were presented in a random order. The whole task lasted approximately 8 min.

### Behavioral Analysis

Trials with no response within the RT window were treated as invalid trials. In addition, trials with an RT exceeding two times the standard deviation (±2 SD) of the mean RT in each condition were considered outliers. Both invalid and outlier trials were excluded from following analyses. The mean and *SD* of RTs for each condition were calculated based on the remaining trials for each participant. The error rate (ER) for each condition was computed as the percentage of trials with incorrect responses. Two separate 2 (group: MCI vs. HC) × 2 (congruency: congruent vs. incongruent) repeated-measures ANOVAs were performed on ER and RT. Group served as a between-subjects factor, and congruency served as a within-subjects factor. All the reported values were Greenhouse–Geisser corrected, and Bonferroni correction was used to account for the multiple comparisons problem.

### EEG Recording and Preprocessing

EEG data were recorded with a sampling rate of 500 Hz from 64 Ag–AgCI electrodes placed according to the International 10-20 electrode system (actiCAP, Brain Products GmbH). The online reference electrode was placed on the left mastoid. The impedance was kept lower than 5 kΩ for all electrodes. Offline EEG preprocessing was performed in MATLAB using the EEGLAB toolbox (Delorme and Makeig, 2004). The raw data were re-referenced to the average of all the electrodes and then bandpass filtered between 1 and 30 Hz using the basic FIR filter. Afterwards, continuous EEG data were segmented into 3-s epochs, extending from −2,000 to 1,000 ms relative to the onset of the stimulus. EEG trials were baseline corrected using the prestimulus interval from −2,000 to 0 ms. Epochs with incorrect responses, labeled with predefined markers, were rejected by the program, and those contaminated by gross artifacts (i.e., large muscle activity produced by cough or swallowing) were rejected with visual inspection. An independent component analysis (ICA) was then performed using the runica algorithm (with the default algorithm of binICA and default parameters). Independent components containing artifacts were detected and rejected by a combination of visual inspection and the EEGLAB plugin *ADJUST* (Mognon et al., 2011).

### EEG Data Analysis

Time–frequency distributions (TFDs) of EEG trials were estimated using a windowed Fourier transform (WFT) with a fixed 300-ms Hanning window. We appended 350 zeros to the end of each segment of windowed data, so each data segment has 500 data points, which finally led to a spectral resolution of 1 Hz. For each trial, the WFT algorithm yielded a complex time–frequency estimate *F*(*t*, *f*) at each time–frequency point (*t*, *f*) that extended from −2,000 to 1,000 ms (in steps of 2 ms) in the time domain and from 1 to 30 Hz (in steps of 1 Hz) in the frequency domain. The resulting spectrogram, *P*(*t*, *f*) = |*F*(*t*, *f*)|^2^, represents the signal power as a joint distribution function at each time–frequency point. The prestimulus alpha power (in μV^2^) was estimated by calculating the mean value of all time–frequency points within a region, ranging from −1,000 to −300 ms in the time domain and from 7 to 14 Hz in the frequency domain. The group-level scalp topography of the prestimulus alpha power was computed by spline interpolation.

In both the congruent and incongruent conditions, all trials were divided into two bins based on single-trial RTs (fast and slow trials were the trials whose RTs were less or more than the median RT of all trials, respectively) for each participant.

Two separate 2 (group: MCI vs. HC) × 2 (response speed: fast vs. slow) × 2 (congruency: congruent vs. incongruent) repeated-measures ANOVAs were performed on prestimulus alpha power (extracted from occipital electrodes: Oz, O1, and O2). Group served as a between-subjects factor. Response speed and congruency served as within-subject factors. All the reported values were Greenhouse–Geisser corrected, and Bonferroni correction was used to account for the multiple comparisons problem.

In the congruent and incongruent conditions, Spearman’s rank correlation between prestimulus alpha power and RT were calculated for each participant. Fisher’s *z* scores of a correlation coefficient was estimated using the formula $z=0.5\times \ln\left(\frac{1+r}{1-r}\right)$. A 2 (group: MCI vs. HC) × 2 (congruency: congruent vs. incongruent) repeated-measures ANOVA was performed on Fisher’s *z* score (Zar, 2014). Group served as a between-subjects factor. Congruency served as a within-subject factor. All the reported values were Greenhouse–Geisser corrected, and Bonferroni correction was used to account for the multiple comparisons problem.

In addition, the relationships between electrophysiological features [i.e., prestimulus alpha power and its correlation coefficient (Fisher’s *z* scores) with RT] and cognitive functions (scores of the 10 neuropsychological tests) were computed using the Pearson correlation analysis for older adults with MCI and HCs, respectively. Performing correlation analysis for the MCI and HC groups separately was to avoid the confounding effect due to significant group differences in the Fisher’s *z* score of correlation coefficient and the neuropsychological measures (Miller and Chapman, 2001).

### Support Vector Machine Analysis

To differentiate between older adults with MCI and HCs, we performed an SVM-based classification analysis, including both behavioral measures (i.e., ER and RT in the congruent and incongruent conditions and the flanker effects on ER and RT) and electrophysiological features [i.e., prestimulus alpha power in the combinations of congruency and response speed, the grand mean of prestimulus alpha power across all conditions, the differences in prestimulus alpha power between fast and slow response trials in congruent and incongruent conditions, and the correlations (Fisher’s *z* scores) between prestimulus alpha power and RT in congruent and incongruent conditions]. Classification performance was evaluated using accuracy and the area under a receiver operating characteristic (ROC) curve.

To predict older adults’ performance on neuropsychological tests, we performed an SVM-based regression analysis in which all extracted electrophysiological features were used as the input features [i.e., prestimulus alpha power and its correlation (Fisher’s *z* score) with RT]. Prediction performance was evaluated using the determination coefficient (*R*^2^) and mean squared error (MSE).

**Figure 2:**
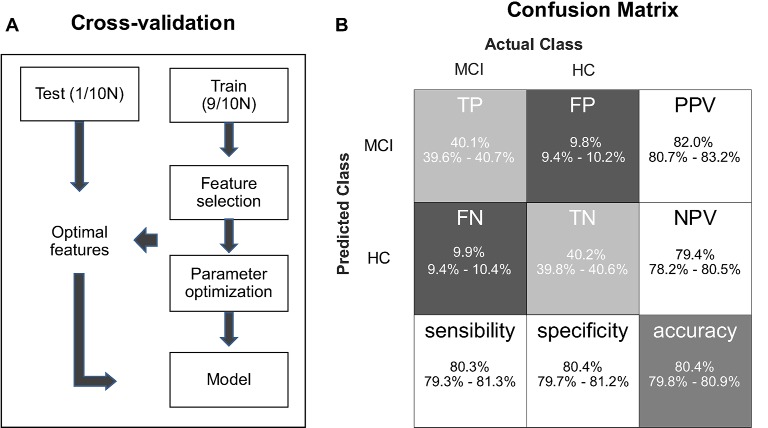
**(A)** A flowchart describing the protocol of cross-validation in support vector machine (SVM) learning. **(B)** The confusion matrix of classification results, which indicates true positive (TP), false positive (FP), positive predictive value (PPV), false negative (FN), true negative (TN), negative predictive value (NPV), sensitivity, specificity, and accuracy.

Please note that in the SVM-based classification and regression analyses, the radial basis function (RBF) kernels with penalty were adopted *via* the LIBSVM toolbox (Chang and Lin, 2011), and all input features were rescaled within the range of 0–1 to minimize interindividual variability (Huang et al., 2013). The enumeration method was used for feature selection, and grid search optimization was used to determine the parameters of cost (*C*, penalty parameter) and gamma (*g*, parameter in the kernel function). We put feature selection and parameter optimization in a nested 10-fold cross-validation, which repeated 100 times. The protocol of the cross validation in SVM analysis is illustrated in Figure 2A.

## Results

### Performance on Neuropsychological Tests

Older adults with MCI showed significantly worse performance on all the neuropsychological tests, except the Stroop test, compared to demographically matched HCs (Table 1).

### Behavioral Results of the Eriksen Flanker Task

For ER, there was no significant main effect of group, *F* < 1. There was a significant main effect of congruency, *F*_(1,54)_ = 9.61, *p* < 0.01, $\eta_{\text{p}}^{2}$ = 0.15. ER in the congruent condition (*M* = 6%, *SD* = 11%) was significantly lower than that in the incongruent condition (*M* = 9%, *SD* = 12%). There was a significant interaction between group and congruency, *F*_(1,54)_ = 4.10, *p* < 0.05, $\eta_{\text{p}}^{2}$ = 0.07. ER in the congruent condition (*M* = 5%, *SD* = 7%) was significantly lower than that in the incongruent condition (*M* = 10%, *SD* = 13%), which was observed in older adults with MCI (*p* < 0.05) rather than in HCs (congruent condition: *M* = 6%, *SD* = 13%; incongruent condition: *M* = 7%, *SD* = 11%; *p* = 0.45).

For RT, there was a significant main effect of congruency, *F*_(1,54)_ = 100.50, *p* < 0.001, $\eta_{\text{p}}^{2}$ = 0.65. RTs were significantly shorter in the congruent condition (*M* = 498 ms, *SD* = 55 ms) than in the incongruent condition (*M* = 538 ms, *SD* = 58 ms). No other significant effect was observed.

### Prestimulus Alpha Power and Its Correlation With RT

**Figure 3:**
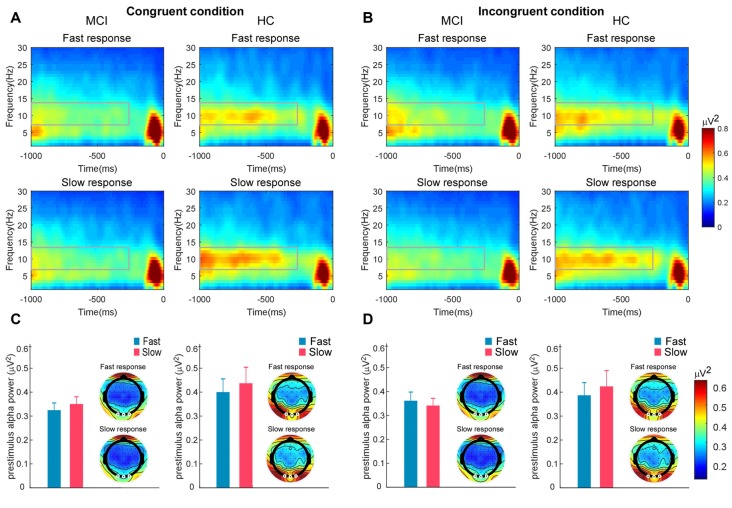
Time–frequency representations highlighted with rectangles illustrate the prestimulus alpha power on the fast and slow trials in the **(A)** congruent and **(B)** incongruent conditions for participants with mild cognitive impairment (MCI) and healthy controls (HCs). The signals were recorded at the occipital electrodes of Oz, O1, and O2. Bar charts illustrate the mean prestimulus alpha power on fast and slow trials in MCI participants and HCs under the **(C)** congruent and **(D)** incongruent conditions. Error bar represents standard error. Scalp topographies beside bar charts illustrate the scalp distributions of prestimulus alpha power across conditions for the MCI and HC groups.

For prestimulus alpha power, there was a significant interaction between response speed and group, *F*_(1,54)_ = 6.28, *p* < 0.05, $\eta_{\text{p}}^{2}$ = 0.10. The prestimulus alpha power on fast trials (*M* = 0.39 μV^2^, *SD* = 0.28 μV^2^) was significantly lower than that on slow trials (*M* = 0.43 μV^2^, *SD* = 0.35 μV^2^) in HCs (*p* < 0.05). However, there was no significant difference between the prestimulus alpha power on fast (*M* = 0.36 μV^2^, *SD* = 0.17 μV^2^) and slow trials (*M* = 0.33 μV^2^, *SD* = 0.15 μV^2^) observed in MCI participants (*p* = 0.18). The alpha power difference between fast and slow trials was significantly larger in the HC group than in the MCI group, *t*_(54)_ = 2.51, *p* < 0.05, with a medium effect size (Cohen’s *d* = 0.72). No other significant effect was observed (Figure 3).

For the Fisher’s *z* score of the correlation coefficient between prestimulus alpha power and RT, there was a significant main effect of group, *F*_(1,54)_ = 5.22, *p* < 0.05, $\eta_{\text{p}}^{2}$ = 0.09. The correlation coefficient was more positive in HCs (*M* = 0.04, *SD* = 0.12) than in older adults with MCI (*M* = −0.03, *SD* = 0.11). The correlation coefficient was significantly higher in the HC group than in the MCI group, *t*_(54)_ = 2.26, *p* < 0.05, with a medium effect size (Cohen’s *d* = 0.62). No other significant effect was observed.

### Relationship Between Electrophysiological Features and Neuropsychological Measures

For older adults with MCI, the prestimulus alpha power was significantly correlated with language function in the congruent condition (BNT: *r* = −0.39, *p* = 0.04) and was marginally significantly correlated with it in the incongruent condition (BNT: *r* = −0.35, *p* = 0.07). For HCs, there was no significant correlation between the prestimulus alpha power and any of the neuropsychological measures.

For older adults with MCI, the Fisher’s *z* scores between prestimulus alpha and RT in the congruent condition were significantly correlated with neuropsychological measures assessed in several different cognitive domains, including executive function (Stroop test: *r* = −0.37, *p* = 0.05), attention (TMT-A: *r* = −0.41, *p* = 0.03), and language (CVFT: *r* = 0.46, *p* = 0.01). For HCs, there was a significant correlation between the Fisher’s *z* score in the congruent condition and the AVLT score reflecting episodic memory (*r* = 0.43, *p* = 0.02). There was no significant correlation between the Fisher’s *z* score in the incongruent condition and any neuropsychological measures for either older adults with MCI or HCs.

### Results of SVM Analysis

For SVM-based classification analysis, we obtained the optimal classifier to be able to differentiate older adults with MCI from HCs with the following five input features: Fisher’s *z* scores in congruent and incongruent conditions, the prestimulus alpha power on slow trials in the congruent condition, the difference in the prestimulus alpha power between fast and slow trials in the congruent condition, and the flanker effect on ER. The trained classifiers had the mean accuracy of 80.4% (95% CI, 79.8–80.9%), the mean area under the ROC curve was 0.77 (95% CI, 0.76–0.78%), the mean sensitivity of 80.3% (95% CI, 79.3–81.3%), the mean specificity of 80.4% (95% CI, 79.7–81.2%), the mean positive predictive values (PPV) of 82.0% (95% CI, 80.7–83.2%), and the mean negative predictive values (NPV) of 79.4% (95% CI, 78.2–80.5%; Figure 2B).

For SVM-based regression analysis, we obtained the optimal models to be able to predict performance on neuropsychological tests, which are summarized in Table 2. The optimal regression model successfully predicted performance on the AVLT delayed recall measure, with an *R*^2^ of 0.24 (95% CI, 0.23–0.25); on the AVLT total score, with an *R*^2^ of 0.28 (95% CI, 0.27–0.29); on the ROCFT Copy, with an *R*^2^ of 0.31 (95% CI, 0.30–0.32); on the Stroop test, with an *R*^2^ of 0.28 (95% CI, 0.27–0.30); and on the BNT, with an *R*^2^ of 0.27 (95% CI, 0.27–0.28; Table 3).

**Table 2:** The input features of optimal models in support vector regression.

Neuropsychological measures	Features
Mini-Mental State Examination (MMSE)	[3,6]
Auditory Verbal Learning Test (delayed recall)	[1,2,4,7,8]
Auditory Verbal Learning Test (total score)	[1,2,4,5,7,8]
Rey–Osterrieth Complex Figure Test (delayed recall)	[5,6,7,9]
Trail Making Test (Part B)	[7,8]
Stroop Test	[2,3,4,5,9]
Trail Making Test (Part A)	[4,8,9]
Symbol Digit Modalities Test	[2,5,6,7,9]
Category Verbal Fluency Tests	[2,4,8]
Boston Naming Test (BNT)	[2,4,5,6,8]
Rey–Osterrieth Complex Figure Test (Copy)	[4,5,8]
Clock Drawing Test	[6]

*[1] Prestimulus alpha power; [2] prestimulus alpha power on fast trials in the congruent condition; [3] prestimulus alpha power on slow trials in the congruent condition; [4] the difference in prestimulus alpha power between fast and slow trials in the congruent condition; [5] prestimulus alpha power on fast trials in the incongruent condition; [6] prestimulus alpha power on slow trials in the incongruent condition; [7] the difference in prestimulus alpha power between fast and slow trials in the incongruent condition; [8] the Fisher’s *z* score in the congruent condition; [9] the Fisher’s *z* score in the incongruent condition*.

**Table 3:** *R*^2^ and mean squared error (MSE) in support vector machine (SVMs).

	*R*^2^ (95% CI)	MSE (95% CI)
Mini-Mental State Examination	0.134 (0.127–0.141)	0.066 (0.066–0.067)
Auditory Verbal Learning Test (delayed recall)	0.241 (0.234–0.249)	0.051 (0.051–0.052)
Auditory Verbal Learning Test (total score)	0.281 (0.273–0.289)	0.034 (0.033–0.034)
Rey–Osterrieth Complex Figure Test (delayed recall)	0.098 (0.092–0.103)	0.056 (0.055–0.057)
Trail Making Test (Part B)	0.087 (0.079–0.094)	0.059 (0.058–0.06)
Stroop Test	0.284 (0.269–0.299)	0.020 (0.020–0.021)
Trail Making Test (Part A)	0.204 (0.198–0.210)	0.035 (0.035–0.036)
Symbol Digit Modalities Test	0.125 (0.118–0.133)	0.038 (0.037–0.038)
Category Verbal Fluency Tests	0.124 (0.118–0.129)	0.023 (0.022–0.023)
Boston Naming Test	0.271 (0.265–0.278)	0.055 (0.054–0.056)
Rey–Osterrieth Complex Figure Test (Copy)	0.308 (0.297–0.320)	0.033 (0.032–0.034)
Clock Drawing Test	0.202 (0.196–0.208)	0.023 (0.0236–0.0241)

## Discussion

This study aimed to investigate whether older adults with MCI would show deficits in preparatory attention compared to those with normal cognition. We focused on the relationship between prestimulus alpha power and RT. There were two findings: (1) There was a significant difference in prestimulus alpha-band power between fast and slow response trials (alpha power fast < alpha power slow) in HCs, while in MCIs, there was no such significant difference; (2) the (Fisher-transformed) correlation coefficients between prestimulus alpha power and RT were significantly higher in HCs (mean correlation coefficient was positive) than in MCIs (mean correlation coefficient was negative). These findings indicate that the relationship between prestimulus alpha power and RT is reduced in older adults with MCI compared to those with normal cognition, suggesting the deficit in preparatory attention in older adults with MCI, which is in line with findings in previous studies using tasks with cues (Levinoff et al., 2005; Tales et al., 2005, 2011).

Our findings extend previous observations of impaired alpha activity in older adults with MCI by demonstrating the abnormal relationship between prestimulus alpha power and RT. Prestimulus alpha activity had less of an impact on RT in older adults with MCI than it does in those with normal cognition. Previous studies have indicated that older adults with MCI show abnormal behavioral performance in terms of RT in the flanker task compared to the performance of those with normal cognition (Wylie et al., 2007; Thurm et al., 2013; Wang et al., 2013). However, there was no significant difference in RT between HCs and older adults with MCI in the present study. Our finding that the reduced correlation between prestimulus alpha power and RT occurred earlier than the abnormal behavior in the flanker task in older adults with MCI suggests that this weakened relationship between prestimulus alpha activity and RT may serve as a feature of impaired preparatory attention at an early stage of MCI.

The literature indicates that preparatory attention is associated with both pre- and post-stimulus inhibition. The deficit in preparatory attention is characterized by the failure of suppressing saccade before onsets of stimuli (Dankner et al., 2017). On the other side, after onsets of stimuli, attentional lapse harms perception, leading to the increase in disruption of irrelevant information, accompanied by enhanced activation of the anterior cingulate cortex (Banich et al., 2000; MacDonald et al., 2000; Botvinick et al., 2001; Weissman et al., 2006). In a Flanker task, the measure of accuracy mainly reflects executive control (Mayr et al., 2003). Thus, the reduced relationship between prestimulus alpha power and RT in older adults with MCI, suggesting a deficit in preparatory attention, might contribute to their reduced accuracy in the task.

Although there was a significant difference in the Fisher’s *z* scores between the two groups, the mean values of the coefficients were small in both groups, suggesting a weak within-subject relationship between prestimulus alpha power and RT. A previous study suggested that the contribution of prestimulus alpha power to reaction time variation is <4% at the within-subject level (Bompas et al., 2015). Such a weak within-subject correlation could also be due to low signal/noise ratios of both signals (i.e., prestimulus alpha power and RT) at a single-trial level.

Fisher’s *z* scores of correlation coefficients between prestimulus alpha power and RT in the congruent condition were associated with some neuropsychological measures (i.e., executive function, attention, and language) in older adults with MCI. Considering that (1) prestimulus alpha power could index the cortical function associated with preparatory attention and top–down attention control (Min and Herrmann, 2007; Romei et al., 2010; Zanto et al., 2011; Knakker et al., 2015; Liu et al., 2016) and (2) Stroop, CVFT, and TMT-A tasks must be performed in a limited amount of time with strong top–down attentional control (Ashendorf et al., 2008; Fan, 2014; Shao et al., 2014), our observation suggests that the decrease in the Fisher-transformed correlation coefficient could serve as a sign of the decline in cognitive function in older adults with MCI.

Previous studies showed that spontaneous alpha activity was significantly lower in amplitude in older adults with MCI than in those with normal cognition (Kwak, 2006; Rossini et al., 2007; Vecchio et al., 2014). In this study, however, no significant difference in prestimulus alpha power between older adults with MCI and HCs was observed. This was probably because the older adults with MCI in this study were recruited from communities, showing less cognitive decline compared to MCI patients recruited from hospitals (Farias et al., 2009).

A deficit in preparatory attention in older adults with MCI was confirmed by the results of machine learning analyses. Prestimulus alpha power and its correlation with RT served as key features in distinguishing older adults with MCI from HCs and in predicting the performance of older adults on some neuropsychological tests (e.g., AVLT delayed recall, AVLT total score, ROCFT Copy, and the Stroop test). In contrast to pure resting-state studies, prestimulus alpha power and its correlation with RT are associated with a particular task and have a specific physiological meaning related to the impaired preparatory attention in older adults. Potentially, these features could serve as important neurophysiological indices to distinguish individuals with MCI from those with normal cognition.

## Data Availability Statement

The datasets generated for this study are available on request to the corresponding author.

## Ethics Statement

The studies involving human participants were reviewed and approved by Institutional Review Board (IRB) of Shenzhen University. The patients/participants provided their written informed consent to participate in this study.

## Author Contributions

PX, YC, and QG designed the experiment. YC, HH, JW, YQ, WF, and QG collected the data. YC and HH analyzed the data. YC, HH, QG, PX, LH, and YL discussed the results and contributed to writing the article.

## Conflict of Interest

The authors declare that the research was conducted in the absence of any commercial or financial relationships that could be construed as a potential conflict of interest.
